# 慢性髓性白血病患者停用酪氨酸激酶抑制剂后停药综合征和心理问题的发生及其影响因素

**DOI:** 10.3760/cma.j.cn121090-20250328-00155

**Published:** 2025-10

**Authors:** 梦瑶 原, 宗儒 李, 小帅 张, 莎莎 赵, 文文 李, 成雷 王, 亚溱 秦, 倩 江

**Affiliations:** 1 北京大学人民医院，北京大学血液病研究所，国家血液系统疾病临床医学研究中心，血液肿瘤细胞和基因治疗北京市重点实验室，北京 100044 Peking University People's Hospital, Peking University Institute of Hematology, National Clinical Research Center for Hematologic Disease, Beijing Key Laboratory of Hematopoietic Stem Cell Transplantation, Beijing 100044, China; 2 北京大学人民医院青岛医院血液中心，青岛 266000 Department of Hematology, Peking University People's Hospital Qingdao Hospital, Qingdao 266000, China

**Keywords:** 白血病，髓性，慢性, 酪氨酸酶抑制剂, 无治疗缓解, 停药综合征, 心理问题, Leukemia, myeloid, chronic, Tyrosine kinase inhibitors, Treatment-free remission, Withdrawal syndrome, Psychological issues

## Abstract

**目的:**

探讨慢性髓性白血病慢性期（CML-CP）患者停用酪氨酸激酶抑制剂（TKI）后停药综合征和心理问题的发生及其影响因素。

**方法:**

回顾性分析北京大学人民医院2012年9月以后停用TKI的CML-CP患者停药前后的临床资料。应用Logistic回归模型探讨停药综合征和心理问题发生的独立影响因素。

**结果:**

共纳入158例患者，女性92例（58％），中位停药年龄50（*IQR* 35～60）岁。停药后中位随访25（*IQR* 11～49）个月，停药4年主要分子学反应（MMR）未丧失生存率为60％（95％ *CI*：51％～70％）。51例（32％）患者在停用TKI中位1.3（*IQR* 0.5～2.0）个月时发生停药综合征。51例患者报告在停用TKI后因担心BCR::ABL1基因水平波动或疾病复发、TKI对胎儿的不良影响和（或）胎儿遗传CML而出现焦虑等心理问题。多因素分析显示，停药时年龄大［纳入TKI治疗时间时，*P*＝0.003；纳入深层分子学反应（DMR）持续时间时，*P*＝0.002］、TKI治疗时间长（*P*＝0.010）和DMR持续时间长（*P*＝0.005）是停用TKI后停药综合征发生的不利因素；受教育程度大学及以上（*P*＝0.010）和因妊娠或药物不良反应停用TKI治疗（*P*＝0.001）与停药后发生心理问题显著相关。停药综合征和心理问题的发生与停药后MMR丧失率无关。

**结论:**

在停用TKI的CML患者中，停药综合征和心理问题较为常见。停药时年龄大、TKI治疗时间长和DMR持续时间长与停药综合征的发生显著相关；受教育水平高、因妊娠或药物不良反应停用TKI与停药后焦虑等心理问题的发生显著相关。

## Abstract (en)

**Objective:**

To investigate the incidence of tyrosine kinase inhibitor（TKI）withdrawal syndrome and psychological issues, and their associated factors, in patients with chronic-phase chronic myeloid leukemia（CML-CP）after TKI discontinuation.

**Methods:**

We retrospectively analyzed the clinical data of CML-CP patients who discontinued TKI therapy at Peking University People's Hospital after September 2012. Logistic regression models were used to identify independent factors associated with the occurrence of TKI withdrawal syndrome and psychological issues.

**Results:**

A total of 158 patients were included, of whom 92（58％）were female. The median age at discontinuation was 50（*IQR*, 35–60）years. With a median follow-up of 25（*IQR*, 11–49）months, the 4-year rate of sustained major molecular response（MMR）was 60％（95％ *CI*: 51％–70％）. Fifty-one（32％）patients experienced TKI withdrawal syndrome at a median of 1.3（*IQR,* 0.5–2.0）months after TKI discontinuation. Fifty-one（32％）patients reported psychological issues such as anxiety. These concerns stemmed from fears of fluctuating BCR::ABL1 levels or disease relapse, and, for those who discontinued TKI for pregnancy, worries about adverse fetal effects and/or the fetus inheriting CML. Multivariable analyses revealed that older age at discontinuation［*P*＝0.003 when adjusting for TKI therapy duration; *P*＝0.002 when adjusting for deep molecular response（DMR）duration］, longer TKI therapy duration（*P*＝0.010）, and longer DMR duration before discontinuation（*P*＝0.005）were significantly associated with a higher risk of TKI withdrawal syndrome; a university degree or higher（*P*＝0.010）and TKI discontinuation due to pregnancy or adverse events（*P*＝0.001）were significantly associated with psychological issues after discontinuation. The occurrence of TKI withdrawal syndrome or psychological issues had no impact on the probability of major molecular response loss after discontinuation.

**Conclusion:**

TKI withdrawal syndrome and psychological issues are common in CML patients who discontinue TKI therapy. Older age at discontinuation and longer TKI therapy duration or DMR duration are significantly associated with TKI withdrawal syndrome. Higher education level and TKI discontinuation due to pregnancy or adverse events are significantly associated with psychological issues.

酪氨酸激酶抑制剂（TKI）显著改善了慢性髓性白血病（CML）患者的预期寿命[1]，无治疗缓解（Treatment-free remission, TFR）逐渐成为新的治疗目标[2]。约50％的TKI治疗患者在获得持续深层分子学反应（Deep molecular response, DMR）后停药可成功实现TFR[3]–[14]。在此背景下，识别预测TFR的因素、探索TKI停药对患者生活质量的影响等成为新的研究重点。

停用伊马替尼后部分患者新发或加重肌肉骨骼疼痛，被定义为停药综合征[15]。随后多项研究显示，20％～30％停用TKI治疗的患者出现肌肉骨骼或关节疼痛、瘙痒、疲劳等症状[10],[13],[16]–[18]。Berger等[16]研究发现，TKI服用时间长、既往有骨关节疼痛病史是停药后短期内出现肌肉骨骼疼痛的风险因素。EURO-SKI研究发现停药时年龄较大、TKI服用时间长是停药后疼痛加重的独立风险因素[19]。此外，TKI停药后还可发生焦虑等心理问题，多因担心BCR::ABL1基因水平波动或疾病复发[18],[20]–[21]，但仅少数停药患者表示曾与医师讨论过如何解决停药后出现的心理问题[22]。TKI停药综合征和心理问题对患者停药后的生活质量产生负面影响，目前在中国CML患者中关于停药综合征和停药后心理问题的研究较少。

了解停药后相关问题的发生及其影响因素有助于促进TKI停药前的医患沟通。因此，本研究回顾性分析我院CML-CP患者TKI停药后停药综合征和心理问题及其对停药后丧失主要分子学反应（MMR）的影响。

## 病例与方法

一、病例

回顾性分析从2006年1月至2020年10月在北京大学人民医院确诊、确诊后6个月内接受TKI作为一线治疗且于2012年后停用TKI的CML-CP连续病例。根据美国国立综合癌症网（NCCN 2024 V2）和欧洲白血病网（ELN）2020版指南[23]–[24]（除TKI停药前治疗失败），纳入标准如下：①停药时年龄≥18岁；②诊断为CML-CP，既往无加速期（CML-AP）或急变期（CML-BP）病史；③停药前接受伊马替尼治疗≥5年或二代TKI（2G-TKI）治疗≥3年；④停药前达持续稳定DMR≥2年，至少通过4次检测确认（每次检测间隔至少3个月）。收集患者社会人口学资料（包括年龄、性别、受教育水平、户籍和婚姻状态）、合并症、ELTS评分、TKI种类、剂量调整和转换、细胞遗传学及分子学检测的结果。TKI停药综合征和心理问题通过常规门诊随访和电话联系的方式回顾性收集。

二、诊断标准、治疗、监测、治疗反应与停药后结局定义

患者诊断、分期、一线治疗选择均参照ELN指南[24]–[27]。TKI治疗期间的监测频率及方法均参考既往研究[28]–[33]。DMR定义为MR^4^（BCR::ABL1^IS^ ≤ 0.01％，ABL转录本>10 000）或MR^4.5^（BCR::ABL1^IS^≤0.003 2％，ABL转录本>32 000）。

停药后监测频率：停药后第1年内每1～2个月进行1次分子学检测；第2年每2个月检测1次；此后每3个月检测1次。失去MMR后，在4周内尽快重启停药前的TKI治疗方案，每月进行分子学检测，直至重新达到MMR，此后每3个月进行1次分子学检测。如果重启TKI治疗3个月后仍未达到MMR，则进行BCR::ABL1激酶结构域突变检测，每月进行监测，持续6个月。

三、停药综合征和心理问题定义

停药综合征定义为停用TKI后加重或新发的肌肉骨骼或关节疼痛，以及其他症状，如瘙痒和疲劳等。参照美国国立卫生研究院/美国国家癌症研究所公布的常见毒性标准CTCAE 5.0进行分级，具体如下：1级：轻度疼痛；2级：中度疼痛，工具性日常生活活动受限；3级：重度疼痛；日常生活自理能力受限。

心理问题定义为停用TKI后因各种原因新发的焦虑等不良情绪。

四、随访

随访方式为门诊或电话联系，截止时间为2025年1月。

五、统计学处理

患者基线信息包括人口学资料和停药时临床特征采用描述性统计分析，分类变量用频数和频率进行描述，连续变量用中位数（范围）或中位数（*IQR*）进行描述。针对连续变量采用受试者工作特征曲线（ROC）（对于二分类结局变量）或X-tile软件（对于时间依赖性结局变量）确定其最佳截断值。对于停药后结局采用Kaplan-Meier生存曲线分析，并应用Log-rank检验进行组间比较；将患者社会人口学因素和停药时临床特征进行单因素分析时，*P*<0.2的变量纳入Logistic回归模型（停药综合征和心理问题的发生）或Cox回归模型（停药后结局）进行多因素分析。根据多因素分析结果，将影响因素作为分组变量，对患者进行危险度评分。*P*<0.05为差异有统计学意义。采用SPSS 26.0、R 4.0.2及GraphPad Prism 9软件进行统计分析、绘图。

## 结果

一、患者基线特征

本研究纳入在北京大学人民医院接受TKI治疗、满足停药标准而终止TKI治疗的158例患者。女性92例（58％），中位诊断年龄41（*IQR* 28～50）岁，中位停药年龄50（*IQR* 35～60）岁。伴合并症25例（22％）。122例患者确诊时具有ELTS评分，包括低危90例（74％）、中危21例（17％）、高危11例（9％）。患者一线TKI治疗药物分别为伊马替尼125例（79％）、尼洛替尼27例（17％）、达沙替尼5例（3％）、氟马替尼1例（1％）。停药前继续维持一线TKI治疗患者128例（81％），30例患者接受了2种或 ≥ 3种TKI治疗，换药原因为治疗失败13例（43％）（包括未达到TKI治疗反应里程碑7例、获得ABL突变5例、失去完全细胞遗传学反应1例）、不耐受6例（20％）、个人意愿11例（37％）。患者停药前使用TKI药物分别为伊马替尼114例（72％）、尼洛替尼30例（19％）、达沙替尼13例（8％）、普那替尼1例（1％）。停药前经历药物剂量减少患者58例（37％），中位减量持续时间为4.5（*IQR* 2.8～6.2）年。

停药前TKI中位治疗时间98（*IQR* 75～131）个月，DMR中位持续时间71（*IQR* 46～101）个月。136例（86％）患者在停药前达到MR^4.5^。停药原因分别为：因持续稳定DMR尝试TFR 72例（46％）、药物不良反应48例（30％）、计划或意外妊娠38例（24％）。158例停药患者的基线特征见表1。

**表1:** 158例慢性髓性白血病停药患者的基线特征

特征	数值
诊断年龄［岁，*M*（*IQR*）］	41（28～50）
停药年龄［岁，*M*（*IQR*）］	50（35～60）
女性［例（％）］	92（58）
户籍［例（％）］	
城镇	117（74）
农村	41（26）
婚姻状态［例（％）］	
已婚	137（87）
未婚	15（10）
离异/丧偶	6（4）
受教育水平［例（％）］	
初中及以下	33（21）
高中	40（25）
大学及以上	85（54）
ELTS评分［例（％）］	
低危	90（57）
中危	21（13）
高危	11（7）
不详	36（23）
合并症［例（％）］	35（22）
一线TKI［例（％）］	
伊马替尼	125（79）
尼洛替尼	27（17）
达沙替尼	5（3）
氟马替尼	1（1）
停药前TKI［例（％）］	
伊马替尼	114（72）
尼洛替尼	30（19）
达沙替尼	13（8）
普纳替尼	1（1）
停药前减量［例（％）］	58（37）
停药前治疗失败史［例（％）］	13（8）
停药前达到MR^4.5^［例（％）］	
是	136（86）
否	12（8）
不详	10（6）
停药原因［例（％）］	
持续稳定DMR	72（46）
药物不良反应	48（30）
意外或计划妊娠	38（24）
TKI治疗时间［月，*M*（*IQR*）］	98（75～131）
治疗至达到DMR时间［月，*M*（*IQR*）］	21（12～39）
DMR持续时间［月，*M（IQR）*］	71（46～101）

**注** DMR：深层分子学反应；TKI：酪氨酸激酶抑制剂；MR^4.5^：分子学反应4.5

二、停药后结局

停药后中位随访25（*IQR* 11～49）个月，58例（37％）患者在停药后中位4（*IQR* 3～7）个月丧失MR^4^；51例（32％）患者在停药后中位4（*IQR* 3～8）个月丧失MMR，其中30例患者在停药后6个月内丧失MMR，20例停药后6～24个月内丧失MMR，1例患者在停药后第46个月丧失MMR。停药后1、2、3、4年MMR未丧失生存率分别为67％（95％ *CI*：60％～75％）、63％（95％ *CI*：55％～72％）、63％（95％ *CI*：55％～72％）、60％（95％ *CI*：51％～70％）。48例因药物不良反应停药的患者停药后其原有的不良反应均有不同程度缓解。

51例丧失MMR的患者均重启TKI治疗，其中44例（86％）患者重新服用停药前TKI药物。截至末次随访，43例（91％）患者在重启TKI治疗中位2（*IQR* 1～4）个月重新达到MMR。此外，10例未丧失MMR的患者重启TKI治疗，原因如下：丧失MR^4^ 1例、出现3级骨骼肌肉或关节疼痛症状1例、分娩结束5例和担心丧失MR^4^ 3例。

在因计划或意外妊娠而停用TKI治疗的38例患者中，18例（47％）患者在停药中位4（*IQR* 3～8）个月丧失MMR。这18例患者在分娩后均重启TKI治疗，重启治疗中位3（*IQR* 2～8）个月重新达到MMR。在未丧失MMR的20例患者中，15例在分娩后继续停药。

三、停药综合征

1. 停药综合征的发生情况：51例（32％）患者在停用TKI中位1.3（*IQR* 0.5～2.0）个月发生停药综合征。47例（92％）出现1～3级的肌肉骨骼或关节疼痛症状，中位持续时间为6（*IQR* 3～12）个月；服用解热镇痛药物患者15例（32％）。此外，1例患者因3级肌肉骨骼或关节疼痛，在未经医师建议的情况下重启TKI治疗。8例（17％）患者停药后出现了1级瘙痒、疲劳等症状；3例（6％）同时出现了肌肉骨骼或关节疼痛和其他症状。32例患者停药综合征的持续时间≥6个月和（或）严重程度 ≥ 2级。

在因计划或意外妊娠而停用TKI治疗的38例患者中，4例（11％）出现了1～2级肌肉骨骼或关节疼痛症状，其中2例患者需服用解热镇痛药物。

2. 停药综合征发生的影响因素分析：将人口学特征、ELTS危险度、停药时年龄、停药前TKI应用情况、停药前TKI治疗时间、TKI治疗至达到稳定DMR时间、DMR持续时间等因素纳入单因素分析，*P*<0.2的变量纳入多因素分析。由于停药前TKI治疗时间和DMR持续时间之间存在共线性，我们将这两个协变量分别纳入多因素分析中，构建两个独立的Logistic回归模型。在纳入TKI治疗时间进行多因素分析，结果显示停药年龄大（*OR*＝1.5,95％*CI*：1.1～1.9；*P*＝0.003）和TKI治疗时间长（*OR*＝2.9,95％*CI*：1.3～6.7；*P*＝0.010）是停用TKI后停药综合征发生的不利因素（表2）；纳入DMR持续时间进行多因素分析，结果显示停药年龄大（*OR*＝1.5,95％*CI*：1.2～1.9；*P*＝0.002）和DMR持续时间长（*OR*＝3.4,95％*CI*：1.5～8.1；*P*＝0.005）是停用TKI后停药综合征发生的不利因素（表2）。停药前使用TKI的种类、停药前是否减少剂量或突然停用、性别等因素均与TKI停药综合征的发生无关。

**表2:** 影响慢性髓性白血病慢性期患者停药综合征和心理问题发生的多因素分析结果

影响因素	停药综合征		心理问题	
	*OR*（95％*CI*）	*P*值	*OR*（95％*CI*）	*P*值
纳入TKI治疗时间				
停药时年龄（每增加10岁）	1.5（1.1～1.9）	0.003		
大学及以上受教育水平（大学以下受教育水平为参考）			2.6（1.3～5.5）	0.010
因药物不良反应或妊娠停药（持续稳定DMR为参考）			3.7（1.8～8.0）	0.001
TKI治疗时间（每增加100个月）	2.9（1.3～6.7）	0.010		
纳入停药前DMR持续时间				
停药时年龄（每增加10岁）	1.5（1.2～1.9）	0.002		
大学及以上受教育水平（大学以下受教育水平为参考）			2.6（1.3～5.5）	0.010
因药物不良反应或妊娠停药（持续稳定DMR为参考）			3.7（1.8～8.0）	0.001
停药前DMR持续时间（每增加100个月）	3.4（1.5～8.1）	0.005		

**注** DMR：深层分子学反应；TKI：酪氨酸激酶抑制剂

以伊马替尼治疗为主的122例（77％）患者在停药前的TKI治疗时间（*P*＝0.016）和DMR持续时间（*P*＝0.020）均显著长于以2G-TKI治疗为主的36例（23％）患者。因此，我们在两组患者中分别进行分析。这两组患者的TKI停药综合征发生率相似（33％对31％，*P*＝0.800）。

在以伊马替尼治疗为主的患者中进行多因素分析，结果显示，无论纳入TKI治疗时间还是DMR持续时间，停药年龄大（*OR*＝1.6,95％*CI*：1.2～2.2；*P*＝0.002或*OR*＝1.6,95％*CI*：1.2～2.2；*P*＝0.001）、TKI治疗时间长（*OR*＝2.5,95％*CI*：1.0～6.2；*P*＝0.052）或DMR持续时间长（*OR*＝2.8,95％*CI*：1.1～7.2；*P*＝0.030）是停用TKI后停药综合征发生的不利因素。通过ROC分析对连续变量选取最大约登指数所对应值为截断值，分别为：停药时年龄46岁、TKI治疗时间103个月、DMR持续时间70个月。多因素分析结果显示，这些分类变量仍与TKI停药综合征的发生显著相关。

根据多因素分析识别出的TKI停药综合征发生的独立影响因素（停药时年龄、TKI治疗时间、DMR持续时间）将患者分为3个亚组：低危组（停药时年龄<46岁、DMR持续时间<70个月且TKI治疗时间<103个月，32例，26％）、中危组（①停药时年龄<46岁、DMR持续时间<70个月且TKI治疗时间≥103个月；②停药时年龄<46岁，DMR持续时间≥ 70个月；③停药时年龄≥46岁，DMR持续时间<70个月，42例，34％）、高危组（停药时年龄≥46岁，DMR持续时间≥70个月，48例，39％）。各组间停药综合征的发生率分别为6％、24％和58％（*P*<0.001）。

由于以2G-TKI持续治疗为主的患者数量有限，未进行多因素分析。单因素分析结果显示，TKI治疗时间长（*P*＝0.079）和DMR持续时间长（*P*＝0.025）是停药综合征发生的不利因素。通过ROC分析对连续变量选取最大约登指数所对应值为截断值，分别是：TKI治疗时间99个月、DMR持续时间89个月。

根据所识别出的危险因素将患者分组，合并停药综合征发生率差异无统计学意义的亚组，将患者分为低危组（DMR持续时间<89个月，30例，83％）和高危组（DMR持续时间≥89个月，6例，17％）。停药综合征的发生率分别为20％和83％（*P*＝0.003）。

四、停药后心理问题

1. 心理问题的发生情况：51例患者报告在停用TKI后存在焦虑相关心理问题。在因计划或意外妊娠而停用TKI治疗的38例患者中，18例（47％）因担心BCR::ABL1基因水平波动、TKI对胎儿的不良影响和（或）胎儿遗传CML而出现焦虑等心理问题。因药物不良反应停用TKI治疗的48例患者中，18例（38％）因担心BCR::ABL1基因水平波动或疾病复发而出现焦虑等心理问题；因持续稳定DMR尝试TFR的72例患者中，15例（21％）也因类似担忧而出现心理问题。

2. 心理问题发生的影响因素分析：将人口学特征、ELTS危险度、停药时年龄、停药前TKI应用情况、停药原因、停药前TKI治疗时间、TKI治疗至达到稳定DMR时间、DMR持续时间等因素纳入单因素分析，*P*<0.2的变量纳入多因素分析。多因素分析结果显示，受教育程度大学及以上（*OR*＝2.6,95％*CI*：1.3～5.5；*P*＝0.010）和因妊娠或药物不良反应停用TKI治疗（*OR*＝3.7,95％*CI*：1.8～8.0；*P*＝0.001）与停药后发生心理问题显著相关（表2）。

五、停药后MMR丧失的影响因素分析

将人口学特征、ELTS危险度、停药时年龄、停药前TKI应用情况、停药原因、停药前TKI治疗时间、TKI治疗至达到稳定DMR时间、DMR持续时间、停药后停药综合征是否发生、心理问题是否出现等因素纳入单因素分析，*P*<0.2的变量纳入多因素分析。多因素分析结果显示，TKI治疗时间短（*HR*＝0.3,95％*CI*：0.2～0.7；*P*＝0.004）和停药前DMR持续时间短（*HR*＝0.2,95％*CI*：0.1～0.5；*P*＝0.001）与停药后丧失MMR显著相关。停药后停药综合征或心理问题的发生、停药前是否减少剂量或突然停用、停药前是否达到MR^4.5^、停药原因等因素均与MMR丧失无关。

在整体患者中，运用X-tile软件对连续变量（DMR持续时间和TKI治疗时间）确定截断值使其变为三分类变量：DMR持续时间46个月、69个月；TKI治疗时间56个月、114个月。根据MMR丧失的独立影响因素将患者分为8个亚组，考虑到临床可操作性，合并差异无统计学意义的组别，最终将患者分为3个亚组：低危组（TKI治疗时间≥114个月且DMR持续时间≥46个月，54例，34％）、中危组（TKI治疗时间56～114个月且DMR持续时间≥46个月，57例，36％）、高危组（TKI治疗时间<114个月，或TKI治疗时间≥56个月且DMR持续时间<46个月，47例，30％）。各组间4年MMR未丧失率分别为83％（95％*CI*：73％～95％）、55％（95％*CI*：41％～74％）和44％（95％*CI*：31％～62％），差异具有统计学意义（*P*<0.001）（图1）。

**图1:**
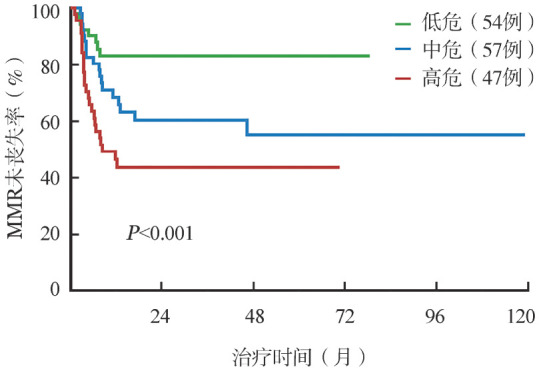
158例慢性髓性白血病慢性期患者酪氨酸激酶抑制剂（TKI）停药后主要分子学反应（MMR）未丧失率 **注** 低危：TKI治疗时间≥114个月且DMR持续时间≥46个月；中危：TKI治疗时间56～114个月且DMR持续时间≥46个月；高危：TKI治疗时间<114个月，或TKI治疗时间≥56个月且DMR持续时间<46个月

此外，各组间停药综合征的发生率分别为48％、28％和19％，差异具有统计学意义（*P*＝0.006）；但在心理问题的发生率上差异并无统计学意义（*P*＝0.145）。

在伊马替尼持续治疗为主和2G-TKI持续治疗为主的患者中分别进行分析。伊马替尼持续治疗组与整体患者结果相似。但2G-TKI持续治疗组的单因素分析中，未得出与MMR丧失相关的影响因素。

## 讨论

本研究探讨了CML患者停用TKI后停药综合征和心理问题的发生情况及影响因素分析。与既往研究发现一致[16],[19]，本研究中32％患者停用TKI后出现停药综合征，停药时年龄大、TKI治疗时间长和DMR持续时间长与TKI停药综合征发生显著相关。值得注意的是，高危组中约60％的患者可能会出现停药综合征。

本研究中32％停药患者在停用TKI后发生心理问题，分析得出结论，受教育程度较高、因妊娠或药物不良反应停药的患者在停用TKI后发生心理问题的风险更高。受教育程度较高的患者对疾病有更深刻的理解，自我管理能力更强，对基因水平的潜在波动或疾病复发更为警惕，更易出现焦虑等心理问题。本研究中，47％因妊娠而停用TKI治疗的患者报告出现了心理问题。孕期本身是一个特殊的生理和心理阶段，孕妇会经历显著的生理、心理和社会变化，这些变化可能导致孕妇情绪波动，使妊娠患者更易出现焦虑等心理问题[34]。此外，担心疾病复发以及TKI药物对胎儿的潜在影响可能进一步加重孕妇的心理负担，从而加剧妊娠患者的心理问题。在临床实践中，医师应特别关注这些高风险人群在停药后的心理状态，及时给予必要的心理支持和干预。

既往一项从患者角度开展的研究表明，在停药过程中，仅7％的患者被询问是否需要心理支持；而在出现停药综合征的患者中，40％患者表示医师未能充分支持并管理其停药后的不适[22]。停药综合征的发生机制尚未完全明确，研究提示其可能涉及多因素协同作用。长期TKI治疗可能通过抑制酪氨酸激酶活性破坏免疫稳态，停药后免疫恢复可能引发炎症因子级联释放，导致骨骼肌肉疼痛等症状[35]。其次，TKI对c-Kit受体的抑制作用可能干扰痛觉调节通路，停药后受体功能恢复可能诱发特征性骨骼肌肉疼痛，动物实验已观察到热痛敏感性改变[36]。此外，TKI的免疫调节作用可能参与局部炎症反应，但多数患者缺乏典型炎症标志物（如C反应蛋白）升高，提示潜在的非经典炎症通路或免疫失衡机制[15]。值得注意的是，停药综合征的临床表现存在种族异质性，例如亚洲患者瘙痒症状发生率显著高于欧洲人群，可能与遗传背景或免疫微环境差异相关[8],[16]。EURO-SKI研究结果发现，60岁及以上的患者在健康相关生活质量（HRQoL）和症状的多个维度上，较年轻患者改善幅度较小[19]。本研究发现，老年患者在停药后更易出现停药综合征，可能与免疫功能衰退、骨代谢异常相关[37]；此外，随着年龄增加，患者肝肾功能减退导致药物清除延迟，TKI长期蓄积后停药引发体内药物浓度骤减，加剧代谢适应性紊乱[38]。多项研究证实，TKI治疗持续时间是停药后维持MMR的独立预测因素[9],[13],[39]，但本研究发现TKI治疗持续时间越长，停药后患者发生停药综合征的风险越高。未来应优化靶向治疗策略，快速、深刻地诱导分子学反应，在确保治疗获益的前提下，缩短治疗时长，个体化治疗以平衡疗效与安全性。

除TKI服用时间长，DMR持续时间长、一线TKI治疗期间BCR::ABL1基因水平快速下降等预测TFR的变量外[3],[7]–[9],[13],[40]，研究发现停用伊马替尼后停药综合征的发生与较低的分子学复发率相关[8]。尽管我们在丧失MMR的低风险组中观察到停药综合征的发生率最高，但在多因素分析中并未发现停药综合征的发生与停药后MMR丧失之间存在显著相关性。

本研究有以下局限性：①为单中心、回顾性研究；②样本量较小；③本研究基于患者的自我报告数据，可能存在回忆偏倚和自我报告偏倚，更精准的CML停药后患者报告结局需通过大样本、多中心、前瞻性研究进一步探索；④本研究中有四分之一患者因妊娠停用TKI。

总之，本研究发现在停用TKI的CML患者中，停药综合征和心理问题较为常见。停药时年龄大、TKI治疗时间长和DMR持续时间长与停药综合征的发生显著相关；受教育水平高、因妊娠或药物不良反应停用TKI与停药后焦虑等心理问题的发生显著相关。停药综合征和心理问题的发生与停药后MMR丧失无关。临床医师应重点关注可能发生停药综合征及心理问题的高危人群，在停用TKI治疗前确保充分的医患沟通，在停药后不仅需密切监测患者病情，还应关注患者的心理健康状态和生活质量变化，及时提供专业支持与指导。
